# The differentiated roles of health in the transition from work to retirement – conceptual and methodological challenges and avenues for future research

**DOI:** 10.5271/sjweh.4017

**Published:** 2022-04-29

**Authors:** Hans Martin Hasselhorn, Taina Leinonen, Ute Bültmann, Ingrid Sivesind Mehlum, Jean-Baptist du Prel, Sibel Kiran, Nicole Majery, Svetlana Solovieva, Astrid de Wind

**Affiliations:** 1 Department of Occupational Health Science, University of Wuppertal, Germany; 2 Finnish Institute of Occupational Health, Helsinki, Finland; 3 Department of Health Sciences, Community and Occupational Medicine, University Medical Center Groningen, University of Groningen, Groningen, The Netherlands; 4 National Institute of Occupational Health (STAMI), Oslo; Institute of Health and Society, University of Oslo, Norway; 5 Institute of Public Health, Department of Occupational Health and Safety, Hacettepe University, Ankara, Turkey; 6 Multisectoral Occupational Health Service, Luxembourg, Luxembourg; 7 Amsterdam UMC, University of Amsterdam, Department of Public and Occupational Health, Amsterdam Public Health Research Institute, Amsterdam, The Netherlands

**Keywords:** concept, employment, indicator, pension, recommendation, work–retirement transition

## Abstract

**Objectives:**

The aim of this discussion paper is to (i) identify the differentiated roles of health in the work–retirement transition, and, with respect to these, (ii) highlight topics related to conceptual and methodological problems and challenges in research, and (iii) present avenues for future research.

**Methods:**

This discussion paper summarizes an OMEGA-NET working group discussion ongoing from November 2018 to September 2021 with face-to-face and online meetings as well as a written online discourse.

**Results:**

‘Health’ and ‘retirement’ are ambiguous concepts. With respect to both, in retirement research, the choice of concept and indicator influences the findings. In addition, the impact of health on retirement is not necessarily a direct one, but can be influenced by further factors such as the ability, motivation and opportunity to work. The strong overall association of poor health with retiring early (path 1) bears the risk of masking distinct and deviating mechanisms in subgroups. In fact, there is evidence that also good health may lead to early retirement (path 2), while both poor health (path 3) and good health (path 4) may also make people retire later.

**Conclusions:**

An increased awareness of the differentiated roles that health may have in the work–retirement transition as outlined in this discussion paper may support research to address questions relevant for policy and practice and increase the impact of research. Recommendations for occupational health and social research are given.

To meet demographic challenges, policies in most Western countries are – for 20 years – designed to extend working lives. Common approaches are restricting access to early exit routes, increasing statutory retirement age and/or rewarding working longer (1). Yet, as concerns about the financial sustainability of pension systems and retirement income adequacy remain (2), it may be assumed that this political trend has not come to an end. While some countries, eg, Finland, Denmark, The Netherlands, Italy and Greece, have automatized an increase of the statutory retirement age by indexing it to the increase in the life expectancy (3), others would be expected to consider further increases of pensionable age. The trends and future perspectives on extended working lives raise a number of key challenges for social and occupational health research and how research adequately informs policy makers. A robust collection of evidence is required to explore whether and under which circumstances future generations of older workers will be willing, able and enabled to work longer. In this discussion paper, the term “work” is understood as paid work both as an employee and as a self-employed person.

A common notion is that the crucial factor for the economic and social development of our ageing societies will be the health of the older working population. National and European policies are emphasizing the relevance of preventive health programmes for prolonging healthy and productive life years (4). Also, retirement research has focused on “health” as a core determinant of staying on the job – or leaving early [see, for example, the review by Oksanen & Virtanen (5)]. Usually, the understanding of this issue is straightforward: poor health leads to early exit from work. While supported by substantial evidence (6–8), this understanding falls short in reflecting the diversity of roles which health may play in the transition from work to retirement. A comprehensive understanding of the multiple roles of health in the work–retirement transition, might advance the awareness for specific vulnerable groups in this transition. Moreover, a further elaboration on the role of health may put health in the context of further factors shaping the work–retirement transition on the individual level (micro-level, eg, work ability, job satisfaction, motivation to keep working, wealth), the organizational level (meso-level, eg, human resource practices, working conditions, work accommodations, leadership quality) and also on the national level (macro-level, e.g. labor market, social legislation and regulation, social and economic conditions, social norms). In times of extending working lives, these insights are relevant for the development of interventions on all three levels, the micro-, meso- and macro-level.

OMEGA-NET (omeganetcohorts.eu) is an EU-funded network of occupational health researchers coordinating and integrating occupational health cohorts on an international level. One specific task is to harmonize conceptions relevant to occupational health. In one subgroup, OMEGA-NET participants are investigating different elements of the work–retirement transition in cohort studies with the aim of elaborating and promoting an understanding of crucial conceptualizations and indicators for this transition, ie, “health”, “retirement” and “(healthy) working life expectancy”.

This discussion paper summarizes an OMEGA-NET working group discussion ongoing from November 2018 to September 2021 with face-to-face and online meetings as well as a written online discourse. It focuses on the “health” aspect from an occupational health research perspective and aims to (i) identify the differentiated roles of health in the work–retirement transition, and – with respect to these – (ii) highlight topics related to conceptual and methodological problems and challenges in research and (iii) present avenues for future research. Although we acknowledge that health influences labor market participation throughout working life, our reflections mainly focus on the later part of the working life span, close to the statutory retirement age.

## Part A: The differentiated roles of health in the work–retirement transition – conceptual and methodological problems and challenges

- The association between health and retirement is dependent on the concepts of health and retirement applied.
- The association can further be influenced by factors such as ability, motivation and opportunity to work.
- The notion “poor health leads to early retirement” is too limited, as also good health may lead to early retirement, while both, poor health and good health may also make people retire later.

### What is retirement?

A widespread understanding of “retirement” is the full withdrawal from paid employment. However, the stylized situation of working up to legal retirement age and from one day to the other transitioning into full-time retirement, is rather the exception. Today, retirement often occurs gradually: the – sometimes years long – process from work to full retirement may contain constructs such as bridge employment, second careers, (part-time) disability retirement, unemployment, unsalaried periods, part-time leave, part-time pension, early retirement, working while drawing pension, salaried while not working, working past retirement age, un-retirement and re-retirement, etc (9).

### Two illustrations of work–retirement frameworks and their conceptual consideration of health

Historically, psychologists were among the first to conceptualize the work–retirement transition (eg, 10). In their models, health is usually treated as one among several “individual” or “personal factors” (10–13), often influencing the psychological decision-making process. In recent years, two cohort studies with an explicit occupational health perspective on the transition from work to retirement were established, based on conceptual work–retirement frameworks: the Dutch Study on Transitions in Employment, Ability and Motivation (STREAM, 14) and the German *leben in der Arbeit* (lidA) cohort study on work, age and health (15).

**Figure 1:**
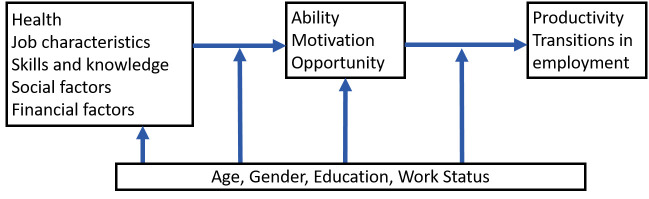
Research framework of Study on Transitions in Employment, Ability and Motivation (STREAM) for the investigation of determinants of transitions in employment and work productivity (14).

**Figure 2:**
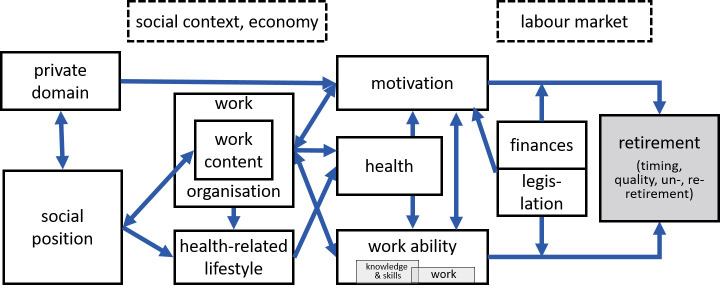
The conceptual framework on work, age and employment for investigating the work–retirement transition of the *leben in der Arbeit* (lidA) German cohort study on work, age and health (16).

In the STREAM research framework, health, job characteristics, skills and knowledge, social factors, and financial factors constitute five domains of potential determinants of transitions in employment (14). The effect of these domains on transitions in employment (and productivity) is mediated by the ability, motivation, and opportunity to work (figure 1). The lidA conceptual framework on work, age and employment combines eleven domains that group core determinants known to influence retirement and indicates their complex interrelations (figure 2). By retirement the authors not only mean the timing of the full exit from working life, but also pathways into retirement as well as un-retirement and re-retirement (16).

Both frameworks are linking together micro-, meso- and macro-level factors. Health, skills, motivation to work, social factors, the financial situation and work ability are individual factors involved, job characteristics and work organization constitute meso-level factors, while opportunity (labor market, economy), legislation and finances may be seen as macro-level factors influencing the health-retirement relation (figures 1 and 2).

While earlier research often focused on the independent effect of health on the timing of work exit, the STREAM and lidA conceptual frameworks suggest a complex interplay of health and other factors that shape the work–retirement transition. In both frameworks, the hypothesized effect of health on the transition from work to retirement is indirect. Both conceptual frameworks make explicit the mediation by the micro-level factors work ability and motivation to work. Further, meso- and macro-level factors influencing the health-retirement relation include the opportunity to work – in the STREAM framework – and legislation and finances in the lidA conceptual framework.

Few studies have empirically investigated the indirect health effects through mediators or moderators as indicated by the conceptual frameworks. De Wind et al (17) found support for the hypothesis that ability and opportunity to work mediate the relation between health and early retirement. De Breij et al (18) showed that in some countries health effects on early work exit were stronger in the lower educated. This is in line with Carr et al (19) who found low educational level and low occupational grade to be associated with health-related early exit from work in seven countries. Further, Leinonen et al (20) have investigated the impact of a Finnish policy reform introducing a lower age limit for statutory retirement. The authors found that workers with better health were more likely to retire earlier due to the reform than workers with poorer health. The first group seemed better able to choose the timing of their retirement according to changes in the normative pension age. In contrast, those with poorer health tended to retire early using any available retirement pathways and less in connection with the statutory pension age.

It is also important to note, that other factors – beyond those described in the two conceptual frameworks – have been identified to influence the health-retirement transition. One such example is the welfare state regime type, reflecting the impact of national welfare and other social institutions. To illustrate, van der Wel et al (22) have found the health effect on non-employment to be consistently lower in the Scandinavian welfare regime than in other regimes. Further, in competing risk analysis of European data, incident poor health was shown to be much stronger associated with receiving disability benefits in Southern European regimes than in Scandinavian, followed by Bismarckian regimes (The Netherlands, France, Germany, Belgium, Austria, Switzerland, 22). Moreover, Brown & Vickerstaff (23) have found job satisfaction, family/caring commitments and the social networks as influential factors for retirement timing limiting the impact of health.

However, both the STREAM and lidA frameworks imply that there is not just one single straightforward but rather multiple ways in which health affects the transition from work to retirement and thereby retirement timing. Based on a simplified conceptualization of the health-retirement association, we exemplify the multitude of paths below.

### Multiple ways of health affecting the timing of retirement

**Figure 3:**
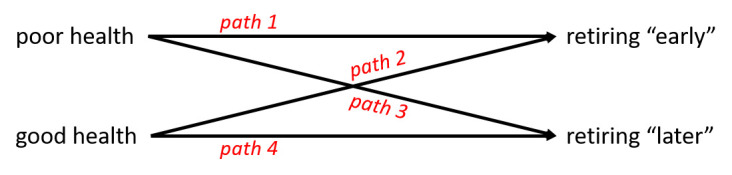
Conceptualization of the health → retirement association, hypothesizing four paths of the impact of health on the timing (early vs. later) of retirement. “Earlier” indicates the exit from paid work prior to statutory retirement age or earlier than the peers in the same professional group. Vice versa: “later” denotes exit after statutory retirement age or later than the peers in the same professional group.

The risk for health problems increases as workers get older (eg, 24, 25). Health problems may include physical and mental health problems and also limitations in physical and cognitive functioning (26). Practice, policy and research tend to be focused on the notion that poor health leads to early exit from work. Yet, not all workers with poor health are leaving employment early and not all who have left early have poor health. In fact, in a representative German sample of persons aged 51–65 years, about one third of all people who were working reported poor health, while among those not working, almost half reported good health (27). Figure 3 illustrates the full range of theoretically possible paths of the health-retirement association among older workers, differentiating four health → retirement paths with respect to the timing of the exit from work. These are discussed below. The authors are aware of the reverse effects, namely that retirement affects health (see for example 5), but these are beyond the scope of this paper.

### Path 1: Poor health is associated with retiring early

In reviews, poor health has been shown to be a strong predictor of early exit from work. Van den Berg et al (6) found significant associations between poor health and early retirement in four of six longitudinal studies. Van Rijn et al (7), when analyzing 29 longitudinal studies, showed that self-reported poor health is a strong predictor of disability pension and to lesser degree of early retirement. Recently, also Scharn et al (8) found poor health to be associated with early exit from work in nine of ten studies included in a systematic review. De Wind et al (28) identified four pathways in which poor health influenced early retirement in a qualitative study based on 30 interviews with early retirees. First, employees may feel unable to work at all due to poor health. Second, health problems may lead to a conception of a future decline in work ability. Third, employees may fear a further decline in health. And fourth, employees with poor health retire early because they feel pushed out by their employer, although they do not experience a reduced work ability. Moreover, older people not working may lack employment opportunities and thus exit work through the unemployment pathway (29), and this may be amplified in case of poor health (28).

### Path 2: Good health is associated with retiring early

Findings from qualitative studies indicate that also good health can lead to early exit from work (23, 28, 30, 31). Based on interviews with older workers who had left work, Pond et al (30) have described a so-called protective pathway, where workers exit work early to protect one’s (still good) health – either because work is perceived as a threat for one’s health or because continued working may not allow for taking care of one’s health. Further, in the maximization of life pathway, people decide to retire while still healthy so that they can fulfil other personal goals. A “health scare” reminding the older worker of his or her own mortality (30) may have preceded this, the health scare may have been induced by an own transient health event or a nearby person’s health failing (23, 28). Good health as a reason for early retirement was often found to be accompanied by the financial opportunity to retire early (28, 31).

However, it should not be overlooked, that many workers with good health are leaving work and employment without giving specific consideration to their own health situation. This may be due to, for example, perceived poor working conditions, a golden handshake, the family situation, leisure aspirations, retirement regulations and norms (eg, 32, 33), and last but not least, unemployment. Obviously, there are many competing pathways into retirement and not all of them are related to health.

### Path 3: Poor health is associated with retiring “later”

Retiring “later” may have several meanings. In this paper, the following understandings are included: working beyond normative retirement ages, such as working longer than peers of the same professional group or working beyond statutory retirement age. Different mechanisms may explain why older workers with poor health retire later: because they can, want, and/or have to work.

*Can work:* Not all workers with poor health experience a reduced work ability. For example, in a nationwide Dutch assessment in 2011, 36% of the workers reported at least one chronic disease and/or handicap; of those, 48% reported no health-related work limitations and 41% only minor limitations. These figures were almost identical in all age groups (34). When work ability is understood as the relation of individual resources to the work demands (35), even workers with poor health may have high work ability. This may occur, for example, when their work demands are low or when the work is adjusted to their individual functioning (see, for example 36) and/or when they have adjustment latitude, ie, the potential to adapt the surrounding to one’s needs, in their private and work environment (37).

*Want to work:* People with poor health may continue to work because they want to. In line with this notion, de Vries et al (37) identified the following motivational drivers for workers with chronic, nonspecific musculoskeletal pain to stay at work: “work as value, therapy, income, and responsibility”. Schmiederer (31) has found that older workers with poor health wanted to continue working for financial reasons and because they felt committed to their profession or their jobs. A precondition for continued working, however, was in all cases, that the work was adapted to their health limitations.

*Have to work:* The idea of older workers with poor health being forced to work longer due to financial reasons is increasingly found in retirement research. Some researchers report that poorer workers are motivated to work longer due to financial factors (38, 39), and that workers with debts are more likely to expect to stop working later (40, 41). In a recent analysis of a representative sample of older workers in Germany, the group with lowest income was found to have poor scores for work and health indicators and higher scores for motivation to work longer. Only in this income group, this motivation was unrelated both to physical and mental health, indicating that the comparably high motivation may be mainly driven by financial imperatives – and irrespective of health (42).

### Path 4: Good health is associated with retiring “later”

Good perceived health can make people retire later. Evidence from qualitative studies indicates that this may be the case. In a British interview study, participants viewed good health as a prerequisite to longer working lives (23). Schmiederer (31) identified workers enjoying the combination of good health and high job satisfaction wanting to work longer. In line with this, Pond et al (30) and also Sewdas et al (43) found that workers regarded their work as a stimulating means to retain good health and to delay presumed retirement-related decline and death.

### Relevance of the health concept used

Health is a term embracing a long list of understandings and definitions (44). While social and health scientists often use self-reported overall health, health conditions or objective health indicators, economists tend to take advantage of register data and construct health indicators, such as ‘health stock’ or a ‘health utility index’ (17, 45). Wikman et al (46) criticize that in public health research, “concepts of ill health are often seen as interchangeable alternatives”. Indeed, there are indications that the researchers’ choice of health indicator matters when investigating the health-retirement association (18). This becomes obvious when taking, for example, the health concept trilogy of “illness”, “disease”, and “sickness”; “illness” refers to the general ill health a person may perceive, “disease” indicates specific diagnosed health conditions and “sickness” relates to social consequences of poor health such as sickness absence. Analysis of Swedish questionnaire and health registry data of the employed population aged 16–65 indicated only a low overlap between the three health concepts (46).

Among the health concepts applied when investigating the work–retirement transition from the social and health science perspectives, the most frequent are chronic diseases in general and perceived general health, followed by mental health and specific diseases, especially musculoskeletal and respiratory diseases and functional limitations, and, finally, register based health claims as administered in the case of hospital admissions, days of treatment and medication use (7, 8). Findings from different studies emphasize the differences in the magnitude of the effects of different health indicators. Van Rijn et al (7) analyzed the influence of different indicators for poor health on the work retirement transition including the exit from paid employment by conducting a meta-analysis of findings from 29 studies. While all health indicators considered in the meta-analysis predicted disability pension, the effect of perceived general health was substantially stronger than that of the other health indicators. This is in line with findings from a Dutch study conducted by van den Berg et al (47). Recent findings by de Breij et al (18) confirm this for Denmark when analyzing prospective data but not for The Netherlands and England, where the effects of functional limitations on disability pension were strongest. With respect to early retirement, only self-perceived health was a significant predictor in the meta-analysis (7). The association of number of chronic diseases with consecutive early retirement was borderline significant, while musculoskeletal and respiratory diseases were clearly insignificant predictors. Unemployment, however, was significantly predicted by all health indicators investigated to similar degree (7). Overall, it may be concluded that – when investigating the work–retirement transition – the choice of the health indicator may influence the findings (18).

### Multiple pathways out of work

When acknowledging the different understandings of retirement (see section “What is retirement?”), it follows that the association of health with retirement also depends on the retirement concept considered. Such concepts are covering a wide range of pathways out of work: In a longitudinal study of 4611 employed persons aged 50–63 years in 11 European countries, van den Berg et al (47) found a population attributable fraction of self-reported poor health to be largest for disability retirement (61%), followed by unemployment (27%) and early retirement (9%). This pattern was largely confirmed by de Breij et al when analyzing data from four European countries (18) and by the previously mentioned systematic literature review and meta-analyses on associations between poor health and exit from paid employment by van Rijn et al (7). People with poor health had an increased likelihood for disability pension, followed by unemployment, and, to a lesser extent, for early retirement (7).

Here, it also needs to be considered that many (early) pathways out of work may be viewed as “communicating vessels” (48, 49). If one way is obstructed, eg, due to limitations in access to disability retirement, other pathways are looked for. In Sweden, for example, blue-collar workers as well as women showed increased claims of early old age pensions following the massively reduced access to disability pension in 2006 (50). Also, earlier evidence from the Netherlands showed that as soon as workers become eligible for an early retirement scheme, this option can be considered a substitute of disability pension (51).

## Part B: Avenues for future research

- Research and policy would benefit from a thorough differentiation of health indicators and retirement outcomes.
- Subgroups with deviating health-retirement patterns, often disadvantaged groups, should not be overlooked when analyzing large samples.
- In times of extended working lives, retirement research and policy should not only focus on the increase of retirement age but also the quality of working life.
- Specific and large cohort studies from a large variety of countries are a basis for good quantitative work-retirement research, preferably combined with qualitative and mixed-methods approaches.

The substantial changes in policy towards extending working lives observed in the past two decades in Europe may continue in times of aging societies. An aspect of high relevance on micro-, meso- and macro-level is, and will remain to be, the role of health. Social and occupational health research will have to inform and advise policy and practice with evidence. The awareness for the differentiated roles that health may have in the work–retirement transition outlined in this discussion paper, may help research to – more than today – ask and investigate the relevant questions and to increase the impact of research. The following recommendations for the consideration of health in work–retirement research will support that.

### Use of different health indicators

Health is an ambiguous concept. In retirement research, the health concept applied and the health indicators used for analysis influence the results. Scientific findings that are based on a particular health indicator may – if this is not made explicit – easily be misunderstood as referring to health in general or to any arbitrary health construct. Researchers should provide a rationale for the health indicators they examine. Particularly when investigating the work–retirement process, it may be appropriate to use different health indicators, this may also include objective indicators. All findings should be discussed in respect to the health indicators used. Further studies are needed to investigate the differential effects of different health concepts and indicators on the transition from work to retirement.

### Combined effects

When researching the work–retirement transition, health cannot be treated as a simple independent variable. Studies should reflect that it usually is a combination of factors, including health, that influence the pathway to retirement – and that this may differ between groups.

### Diversity of retirement outcomes

When investigating the roles of health affecting pathways out of work and timing of retirement, research should account for the diversity of retirement outcomes. Competing exit ways have to be considered and, where appropriate and possible, competing risks analysis should be performed.

### Distinct and deviating mechanisms in subgroups

The strong overall association of poor health with early exit from work bears the risk of masking distinct and deviating mechanisms in subgroups. Such mechanisms have been identified in the substantial amount of qualitative research investigating the process of the work–retirement transition. Quantitative social epidemiology should attempt to identify the mechanisms suggested in qualitative research and take them into consideration when interpreting the findings. Mixed methods research approaches could be a fruitful way to elaborate the complexity of the work–retirement transition.

### Specific longitudinal mixed method studies

More longitudinal population-based studies specific to investigating the work–retirement transition as process are needed. These need to be large to enable the consideration of subgroups, such as specific occupations, those with specific employment trajectories, workers with a migration background or of different income classes. They should repeatedly and in detail assess work and health and cover a broad range of domains as indicated in the conceptual frameworks presented in the paper. Where possible, they should be combined with in-depth qualitative research elements and further enable linkage to register data (eg, employment, retirement, rehabilitation, healthcare utilization).

### Groups getting trapped with poor health in employment

National social policies aimed at extending the working life of the work force may adversely affect those groups who bear the risk of getting trapped with poor health in employment. More research is needed on the effect of such policies on the health of different occupational groups approaching retirement age. While some groups may benefit from these policies (winners), others will turn out as losers, usually those of poor health and low financial resources and in precarious employment (52).

### Quality of working life

The impact of work on the roles of health in the work–retirement transition needs to be considered. In times of extended working life policies, the research attention should not only focus on the increase of the retirement age but also the quality of (working) life and employment opportunities for the different groups of older workers in the last years before retirement. There are concerns that the policies could stagnate in their effects because some people and groups may not be able to respond in the way intended by such policies and reforms (53).

### Monitoring, “stay at work” and “return to work” research

Apart from a thorough work and health-monitoring at higher working age, substantially more “stay at work” research (not least on the effects of work accommodations), “return to work” research and research on the retirement process and regulation is needed. Here, data from large cohorts and registers, but also their linkage, may provide excellent research opportunities.

### Evidence and input from further countries

The predominance of research investigations in the field in only a few countries in Europe is striking. Research on the roles of health in the work–retirement transition would benefit from input from all countries and cross-national comparative approaches – thereby taking into account the underlying administrative and jurisdictional contexts.
